# Supplementary material for: Metaplastic neuromodulation via transcranial direct current stimulation has no effect on corticospinal excitability and neuromuscular fatigue
Source: Exp Brain Res. 2024 Jun 28;242(8):1999–2012. doi: 10.1007/s00221-024-06874-z (PMC11252223; doi:10.1007/s00221-024-06874-z)
Supplement: Supplementary file 1 — Supplementary Material 1 [file 221_2024_6874_MOESM1_ESM.docx]

**Supplementary Material**

|  | **Nothing** | **Stinging** | **Warmth** | **Prickling** | **Dull Headache** | **Tingling** | **Itchy** | **Burning** | **Needling** |
| --- | --- | --- | --- | --- | --- | --- | --- | --- | --- |
| Priming Start | 8 | 1 | 0 | 2 | 0 | 23 | 16 | 9 | 1 |
| Priming Middle | 37 | 0 | 0 | 1 | 1 | 11 | 10 | 0 | 0 |
| Priming End | 43 | 0 | 0 | 1 | 0 | 7 | 8 | 1 | 0 |
| Exercise Start | 7 | 0 | 1 | 1 | 0 | 19 | 16 | 15 | 1 |
| Exercise Middle | 42 | 0 | 0 | 1 | 0 | 8 | 7 | 2 | 0 |
| Exercise End | 52 | 0 | 0 | 1 | 0 | 5 | 1 | 1 | 0 |

Table 1: Frequency table of reported tDCS sensations. The number represents the number of participants reporting the sensation.
